# Thermal characteristics of longitudinal fin with Fourier and non-Fourier heat transfer by Fourier sine transforms

**DOI:** 10.1038/s41598-021-00318-2

**Published:** 2021-12-03

**Authors:** Basma Souayeh, Kashif Ali Abro

**Affiliations:** 1 Department of Physics, College of Science, King Faisal University, PO Box 400, Al Ahsa, 31982 Saudi Arabia; 2 Institute of Ground Water Studies, Faculty of Natural and Agricultural Sciences, University of the Free State, Bloemfontein, South Africa; 3 Department of Basic Sciences and Related Studies, Mehran University of Engineering and Technology, Jamshoro, Pakistan; 4 Laboratory of Fluid Mechanics, Physics Department, Faculty of Science of Tunis, University of Tunis EI Manar, 2092 Tunis, Tunisia

**Keywords:** Fluid dynamics, Energy storage

## Abstract

The quest for high-performance of heat transfer components on the basis of accommodating shapes, smaller weights, lower costs and little volume has significantly diverted the industries for the enhancement of heat dissipation with variable thermal properties of fins. This manuscript proposes the fractional modeling of Fourier and non-Fourier heat transfer of longitudinal fin via non-singular fractional approach. The configuration of longitudinal fin in terms of one dimension is developed for the mathematical model of parabolic and hyperbolic heat transfer equations. By considering the Fourier and non-Fourier heat transfer from longitudinal fin, the mathematical techniques of Fourier sine and Laplace transforms have been invoked. An analytic approach is tackled for handling the governing equation through special functions for the fractionalized parabolic and hyperbolic heat transfer equations in longitudinal fin. For the sake of comparative analysis of parabolic verses hyperbolic heat conduction of fin temperature, we depicted the distinct graphical illustrations; for instance, 2-dimensional graph, bar chart, contour graphs, heat graph, 3-dimensional graphs and column graphs on for the variants of different rheological impacts of longitudinal fin.

## Introduction

The parabolic and hyperbolic heat transfer from longitudinal fin has diverse industrial applications especially in air conditioning, refrigeration and few others. The Mathematical modeling of parabolic and hyperbolic heat transfer equations is not an easy task for investigating fractionalized analytical solution as well as fractionalized numerical solution. This is because temperature distribution of parabolic and hyperbolic heat transfer equations involves temperature-dependent properties^1–8^. In view of preeminent studies investigating the fin problem on Fourier and non-Fourier domain subject to the periodic boundary conditions, a sequential approach has been suggested by Yang in^9^ with direct and inverse analysis based on the Finite difference and modified Newton Raphson methods respectively. Here, a great conformity has been established in results on the basis of accuracy between exact and non-exact techniques. In context with fin problem of non-Fourier thermal conditions, an analytical study has been carried out by Ahmadikia and Rismanian in^4^. They invoked the second law of thermodynamic for hyperbolic model to find temperature field. Moreover, they emphasized the impacts of time relaxation for hyperbolic model only. From mathematical point of view, Aziz et al.^10^ introduced the adjoint conjugate gradient method to evaluate the base temperature in non-Fourier inverse fin problem. The varying thermal conductivity subject to the wavelet collocation method for the nonlinear boundary has been investigated by Singh et al.^11^. Their main objective was to analyze the linear, constant and exponential temperature. Nagarani et al.^12^ observed the temperature distribution from the elliptical and circular annular fin through the empirical structure of computational fluid dynamics and optimizing genetic algorithm. Additionally, they validated the elliptical annular fin through both techniques. The porous fin has been quantified subject to predict the impacts of thermal diffusion and porosity through temperature profile on the fin by Das and Prasad^13^. They invoked differential evolution method to confess the performance with optimized algorithms. For the sake of fin geometry with optimized heat transfer via triangular, convex and concave conditions, analyticity of temperature has been carried out through least square method by Mosayebidorcheh et al.^7^. Recently Jing et al.^14^ invoked spectral element method to determine the non-uniform heat generation with variable temperature for irregular fins in porous structure. For the first time in recent literature, none of fractional model via Atangana-Baleanu differential operator is studied. The authors of this manuscript presented fractional modeling of fin on non-Fourier heat conduction in recent literature. They focused the non-singular time derivative on the mathematical model of fin with non-Fourier heat conduction^15^. In this context, fractional models based on non-singular kernels can have ability to disclose the hidden phenomenon through the memory effects. The distinct studies on fractional models are adhered in this regard as; fractional models based on singular kernel can be viewed in^16–26^, fractional models based on non-singular kernel can be viewed in^27–35^ and fractal-fractional differential and integral models based on singular and local as well as non-singular and non-local kernel can be viewed in^36–46^. Motivating by above discussion, we propose the fractional modeling of Fourier and non-Fourier heat transfer of longitudinal fin via non-singular fractional approach. The configuration of longitudinal fin in terms of one dimension is developed for the mathematical model of parabolic and hyperbolic heat transfer equations. By considering the Fourier and non-Fourier heat transfer from longitudinal fin, the mathematical techniques of Fourier sine and Laplace transforms have been invoked. An analytic approach is tackled for handling the governing equation through special functions for the fractionalized parabolic and hyperbolic heat transfer equations in longitudinal fin. For the sake of comparative analysis of parabolic verses hyperbolic heat conduction of fin temperature, we depicted the distinct graphical illustrations; for instance, 2-dimensional graph, bar chart graph, contour graphs, heat graph, 3-dimensional graphs and column graphs on for the variants of different rheological impacts of longitudinal fin.

## Fractional modeling of Fourier and non-Fourier heat transfer

It is well established fact in literature that Cattaneo and Vernotte^1,2^ proposed the suitable mathematical models of heat conduction for describing the conductive heat transfer in many engineering problems in 1958. Such mathematical models of heat conduction are based on an independently hyperbolic heat conduction model with a finite propagation speed so called non-Fourier model of heat conduction described as:

1 $$\tau \frac{{\partial q\left( {x,t} \right)}}{\partial t} + q\left( {x,t} \right) + k\nabla T\left( {x,t} \right) = 0.$$

**Figure 1:**
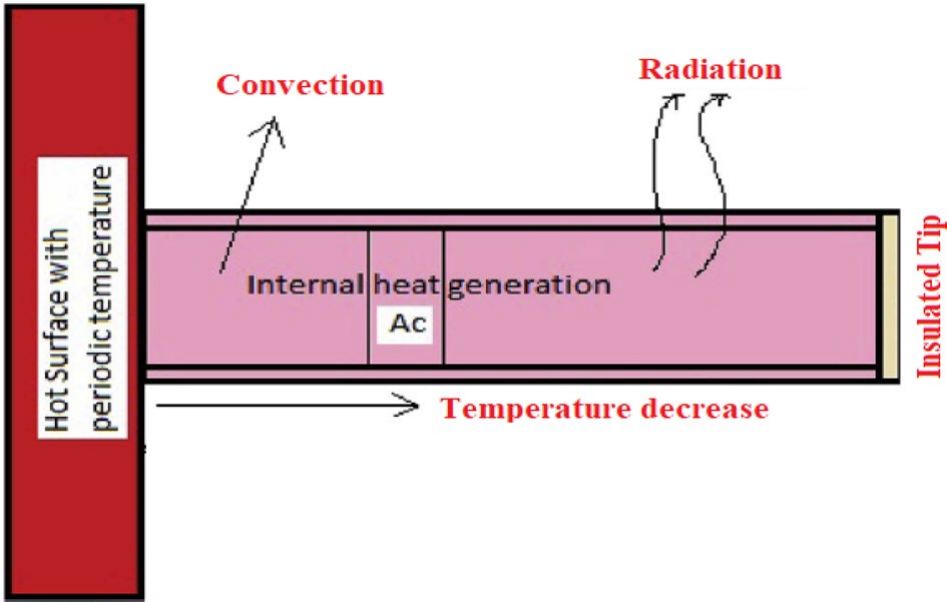
Configuration of longitudinal fin in terms of one dimension.

In this context, a tip of the longitudinal fin is considered adiabatic subject to ratio of the thickness to the length is $\frac{b}{L} < 1$, the cross section area $AC$, relaxation time $\tau$ perimeter $P$, thickness $b$ and length $L$ as depicted in the Fig. 1.

For the sake of heat dissipation in the environment through convection, we treated convective heat transfer coefficient $h$ as constant. Meanwhile, the fin material having specific heat $c$, constant thermal conductivity $k$ and density $\rho$. Additionally, fin contains an internal heat source $q^{*}$ that depends upon the local fin temperature. In order to satisfy the geometry of the longitudinal fin, the following governing differential equations for non-Fourier (hyperbolic) and Fourier (parabolic) heat transfer are:

2 $$\begin{aligned} & \frac{\tau }{c\rho }\frac{{\partial q^{*} }}{\partial t} - \frac{hP}{{\rho Ac^{2} }}\left( {T - T_{\infty } } \right) - \frac{\tau hP}{{\rho Ac^{2} }}\frac{\partial T}{{\partial t}} + \frac{k}{c\rho }\frac{{\partial^{2} T}}{{\partial x^{2} }} - \frac{{q^{*} }}{c\rho } = \left( {\tau \frac{{\partial^{2} }}{{\partial t^{2} }} + \frac{\partial }{\partial t}} \right)T, \\ & \frac{hP}{{\rho Ac^{2} }}\left( {T - T_{\infty } } \right) - \frac{{q^{*} }}{c\rho } = \frac{k}{c\rho }\left( {\frac{{\partial^{2} }}{{\partial x^{2} }} - \frac{\partial }{\partial t}} \right)T. \\ \end{aligned}$$

Equation (2$_{1}$) is governing differential equation for non-Fourier (hyperbolic) heat conduction and equation $\left( {2_{2} } \right)$ is governing differential equation for Fourier (parabolic) heat conduction, in which $q^{*} = q_{\infty }^{*} \left( {\left( {T - T_{\infty } } \right)\varepsilon + 1} \right)$. While, $\tau$ reflects relaxation time in Eq. (2). The relaxation time is a measure of the time that takes heat conduction in the system to be significantly perturbed. From physical aspects, the relaxation time usually means the return of a perturbed heat conduction into equilibrium. The temperature is constant everywhere in fin at $t = 0$ and rate of change of temperature in longitudinal fin with respect to time are subjected to the initial conditions for governing system of partial differential Eqs. (2), we write as:

3 $$T\left( {x,0} \right) = T_{\infty } ,\frac{{\partial T\left( {x,0} \right)}}{\partial t} = 0.$$

Additionally, the base of longitudinal fin to a surface with periodically temperature oscillation is described in the following equation as:

4 $$T_{b} \left( {0,t} \right) = AT_{b,m} \cos \left( {\omega t} \right) + T_{b,m} - AT_{\infty } \cos \left( {\omega t} \right).$$

The functional parameters for Eq. (4) are described in the following Table 1.

**Table 1:** Functional and rheological parameters.

Functional parameter	Description
$\omega$	Frequency of the base temperature
*A*	Dimensionless amplitude of the base temperature
$T_{b,m}$	Mean base temperature
$T_{\infty }$	Ambient temperature
$T_{b}$	Periodic base temperature
*t*	Time variable
$\Omega$	Dimensionless periodicity
$\alpha$	Fractional parameter

In engineering and science, dimensional analysis is the analysis of the relationships between different physical quantities by identifying their base quantities. On introducing the dimensionless variable and similarity criteria, we have defined as:

5 $$\begin{aligned} & y = \frac{x}{L}, t = \frac{kt}{{L^{2} c\rho }},\emptyset = \frac{{T_{b} - T_{\infty } }}{{T_{b,m} - T_{\infty } }},\mathcal{N}_{1}^{2} = \frac{{hpL^{2} }}{KAc},\mathcal{N}_{2} = \frac{{Acq_{\infty }^{*} }}{{\left( {T_{b,m} - T_{\infty } } \right)hp}},\mathcal{N}_{3} = \frac{k\tau }{{L^{2} c\rho }},\Omega = \frac{{\omega L^{2} c\rho }}{k}, \\ & \mathcal{N}_{4} = \varepsilon \left( {T_{b,m} - T_{\infty } } \right),\lambda_{1} = \mathcal{N}_{1}^{2} \left( {1 - \mathcal{N}_{2} \mathcal{N}_{3} } \right),\lambda_{2} = \mathcal{N}_{1}^{2} \mathcal{N}_{2} ,\lambda_{3} = \mathcal{N}_{3} ,\lambda_{4} = 1 + \mathcal{N}_{3} \lambda_{1} . \\ \end{aligned}$$

By introducing Eq. (5) into Eqs. (2–4), the non-fractional parabolic and hyperbolic heat transfers in longitudinal fin’s governing equations are respectively:

6 $$\frac{{\partial \emptyset \left( {y,t} \right)}}{\partial t} = \frac{{\partial^{2} \emptyset \left( {y,t} \right)}}{{\partial y^{2} }} - \lambda_{1} \emptyset \left( {y,t} \right) + \lambda_{2} ,$$

7 $$\lambda_{3} \frac{{\partial^{2} \emptyset \left( {y,t} \right)}}{{\partial t^{2} }} + \lambda_{4} \frac{{\partial \emptyset \left( {y,t} \right)}}{\partial t} = \frac{{\partial^{2} \emptyset \left( {y,t} \right)}}{{\partial y^{2} }} - \lambda_{1} \emptyset \left( {y,t} \right) + \lambda_{2} .$$

Subject to the imposed conditions as:

8 $$\emptyset \left( {x,0} \right) = \frac{{\partial \emptyset \left( {x,0} \right)}}{\partial t} = 0, \emptyset \left( {0,t} \right) = 1 + A\cos \left( {\Omega t} \right).$$

Introducing the AB-fractional differential operator on the governing non-fractional parabolic and hyperbolic heat transfers equations in longitudinal fin’s say (6–7), we define AB-fractional differential operator in Eq. (9) as

9 $$^{AB} \left( {\frac{{\partial^{\alpha } \emptyset }}{{\partial t^{\alpha } }}} \right) = \int\limits_{0}^{t} {\emptyset^{\prime } \left( s \right)\left( {1 - \alpha } \right)^{ - 1} {\text{E}}_{\alpha } \left( {\frac{{ - \alpha \left( {t - s} \right)^{\alpha } }}{1 - \alpha }} \right)ds} .$$

we fractionalized Eqs. (6–7) by invoking Eq. (9), we arrive at

10 $$\frac{{\partial^{\alpha } \emptyset \left( {y,t} \right)}}{{\partial t^{\alpha } }} = \frac{{\partial^{2} \emptyset \left( {y,t} \right)}}{{\partial y^{2} }} - \lambda_{1} \emptyset \left( {y,t} \right) + \lambda_{2} ,$$

11 $$\lambda_{3} \frac{{\partial^{2\alpha } \emptyset \left( {y,t} \right)}}{{\partial t^{2\alpha } }} + \lambda_{4} \frac{{\partial^{\alpha } \emptyset \left( {y,t} \right)}}{{\partial t^{\alpha } }} = \frac{{\partial^{2} \emptyset \left( {y,t} \right)}}{{\partial y^{2} }} - \lambda_{1} \emptyset \left( {y,t} \right) + \lambda_{2} .$$

## Fractional treatment to longitudinal fin with Fourier and non-Fourier heat transfer

**Figure 2:**
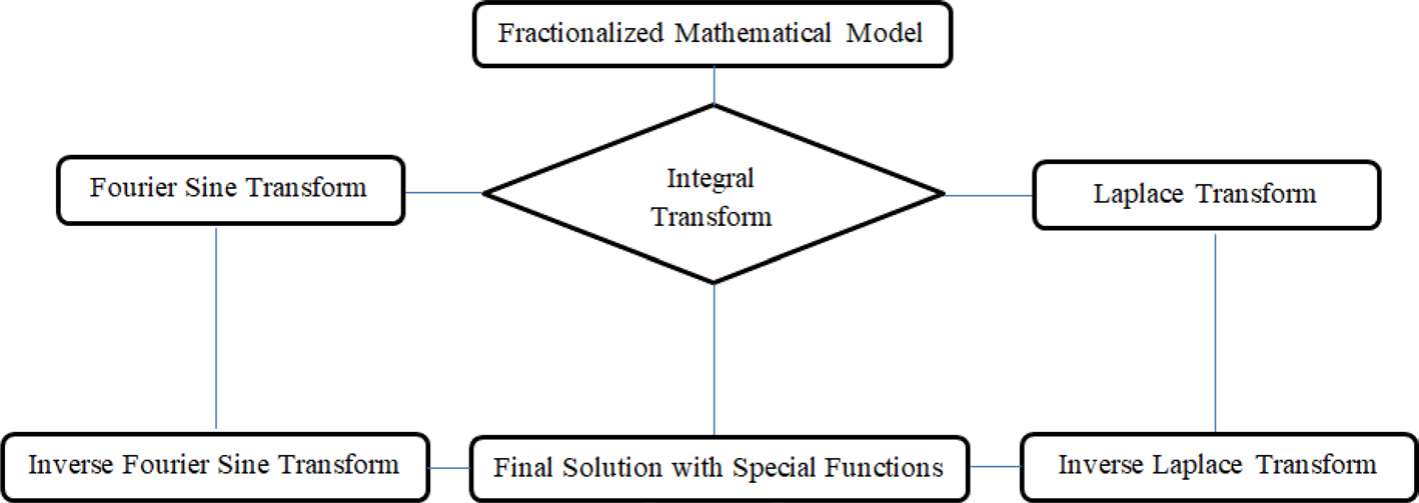
Flow chart for calculation.

Different methodologies based on fractional modeling of longitudinal fin with Fourier and non-Fourier heat transfer have obtained significant role. The well-known methodologies are Laplace transform, control volume method, finite element method, spectral element method, spectral collocation method, differential transform method, response surface method, least square method and several others. Meanwhile, for the sake of deep study, we first time in literature invoked combined Laplace and Fourier sine transforms on the governing non-fractional parabolic and hyperbolic heat transfers equations in longitudinal fin. The flow chart for invoked combined Laplace and Fourier sine transforms on the governing non-fractional parabolic and hyperbolic heat transfers equations in longitudinal fin is sketched as Fig. 2.

### Analytic and fractional treatment to parabolic heat transfer equations in longitudinal fin

Applying Fourier sine transform on Eq. (10), we get

12 $$\frac{{\partial^{\alpha } \emptyset_{s} \left( {\xi ,t} \right)}}{{\partial t^{\alpha } }} = \sqrt {\frac{2}{\pi }} \left( {\frac{{\left( {\xi^{2} + A\xi^{2} cos\Omega t} \right) + \lambda_{2} }}{\xi }} \right) - \left( {\xi^{2} + \lambda_{1} } \right)\emptyset_{s} \left( {\xi ,t} \right).$$

Solving fractional differential Eq. (12) by means of Laplace transform, we arrived at

13 $$\emptyset_{s} \left( {\xi ,q} \right) = \frac{{\sqrt {\frac{2}{\pi }} \left\{ {\left( {\frac{\xi }{q} + \frac{A\xi q}{{q^{2} + \Omega^{2} }}} \right) + \frac{{\lambda_{2} }}{\xi }} \right\}\left( {q^{\alpha } + z_{0} } \right)}}{{q^{\alpha } z_{1} + z_{2} }}.$$

Equation (13) is calculated on the basis of letting parameters as $\beta = \frac{1}{1 - \alpha },z_{0} = \alpha \beta ,z_{1} = \beta + \xi^{2} + \lambda_{1} , z_{2} = \xi^{2} z_{0} + \lambda_{1} z_{0}$. Applying inverse Fourier sine transform on Eq. (13) as:

14 $$\begin{aligned} \overline{\emptyset }\left( {y,q} \right) & = \left( {\frac{1}{q} + \frac{Aq}{{q^{2} + \Omega^{2} }}} \right) - \frac{2}{\pi }\mathop \int \limits_{0}^{\infty } \frac{{sin\left( {y\xi } \right)}}{\xi }\frac{{\left( {z_{1} + \xi^{2} } \right)}}{{z_{1} }}\frac{{\left( {q^{\alpha } + z_{3} } \right)}}{{q\left( {q^{\alpha } + z_{4} } \right)}}d\xi - \frac{2}{\pi }\mathop \int \limits_{0}^{\infty } \frac{{sin\left( {y\xi } \right)}}{\xi }\frac{{\left( {z_{1} + \xi^{2} } \right)}}{{z_{1} }}\frac{Aq}{{q^{2} + \Omega^{2} }} \\ & \quad \times \;\frac{{\left( {q^{\alpha } + z_{3} } \right)}}{{\left( {q^{\alpha } + z_{4} } \right)}}d\xi - \frac{2}{\pi }\mathop \int \limits_{0}^{\infty } sin\left( {y\xi } \right)\frac{{\lambda_{2} }}{{z_{1} }}\frac{{\left( {q^{\alpha } + z_{0} } \right)}}{{\left( {q^{\alpha } + z_{4} } \right)}}d\xi . \\ \end{aligned}$$

The functional parameters for Eq. (14) are $z_{3} = \frac{{z_{2} + \xi^{2} z_{0} }}{{z_{1} + \xi^{2} }},z_{4} = \frac{{z_{2} }}{{z_{1} }}$. Invoking inverse Laplace transform on Eq. (14), we derived final solution in terms of special function as:

15 $$\begin{aligned} \emptyset \left( {y,t} \right) & = 1 + A\cos \left( {\Omega t} \right) - \frac{2}{\pi }\mathop \int \limits_{0}^{\infty } \frac{{sin\left( {y\xi } \right)}}{\xi }\frac{{\left( {z_{1} + \xi^{2} } \right)}}{{z_{1} }}\left\{ {{\text{E}}_{\alpha } \left( {z_{4} t^{\alpha } } \right) + \frac{{z_{3} }}{{z_{4} }}\left( {1 - {\text{E}}_{\alpha } \left( { - z_{4} t^{\alpha } } \right)} \right)} \right\}d\xi - \frac{2A}{\pi }\mathop \int \limits_{0}^{\infty } \frac{{sin\left( {y\xi } \right)}}{\xi } \\ & \quad \times \;\frac{{\left( {z_{1} + \xi^{2} } \right)}}{{z_{1} }}\left\{ {\left( {\delta \left( t \right) - \Omega \sin \left( {\Omega t} \right)*{\text{E}}_{\alpha } \left( {z_{4} t^{\alpha } } \right)} \right) + z_{3} \left( {cos\left( {\Omega t} \right)*t^{\alpha - 1} {\text{E}}_{\alpha ,\alpha } \left( { - z_{4} t^{\alpha } } \right)} \right)} \right\}d\xi \\ & \quad - \;\frac{2}{\pi }\mathop \int \limits_{0}^{\infty } sin\left( {y\xi } \right)\frac{{\lambda_{2} }}{{z_{1} }}\left\{ {\left( {\frac{1}{{t^{\alpha + 1} }}*\frac{{t^{\alpha - 1} {\text{E}}_{\alpha ,\alpha } \left( { - z_{4} t^{\alpha } } \right)}}{{\Gamma \left( { - \alpha } \right)}}} \right) + \frac{{z_{0} t^{\alpha - 1} {\text{E}}_{\alpha ,\alpha } \left( { - z_{4} t^{\alpha } } \right)}}{{z_{4} }}} \right\}d\xi \\ \end{aligned}$$

where the special functions are defined for the fractional parabolic heat transfers equation in longitudinal fin say Eq. (15) as:

16 $$\mathcal{L}^{ - 1} \left( {\frac{{p^{{\text{\rm A}}} }}{{p\left( {p^{{\text{\rm A}}} + {\text{\rm B}}} \right)}}} \right) = {\mathbf{E}}_{{\text{\rm A}}} \left( { - {\text{\rm B}}t^{{\text{\rm A}}} } \right),$$

17 $$\mathcal{L}^{ - 1} \left( {\frac{{\text{\rm B}}}{{p\left( {p^{{\text{\rm A}}} + {\text{\rm B}}} \right)}}} \right) = 1 - {\mathbf{E}}_{{\text{\rm A}}} \left( { - {\text{\rm B}}t^{{\text{\rm A}}} } \right),$$

18 $$\mathcal{L}^{ - 1} \left( {\frac{1}{{\left( {p^{{\text{\rm A}}} + {\text{\rm B}}} \right)}}} \right) = t^{{{\text{\rm A}} - 1}} {\mathbf{E}}_{{{\text{\rm A}},{\text{\rm A}}}} \left( { - {\text{\rm B}}t^{{\text{\rm A}}} } \right).$$

### Analytic and fractional treatment to hyperbolic heat transfer equations in longitudinal fin

Applying Fourier sine transform on Eq. (11), we get

19 $$\left( {\lambda_{3} \frac{{\partial^{\alpha 2} }}{{\partial t^{2\alpha } }} + \lambda_{4} \frac{{\partial^{\alpha } }}{{\partial t^{\alpha } }}} \right)\emptyset_{s} \left( {\xi ,t} \right) = \sqrt {\frac{2}{\pi }} \left\{ {\frac{{\lambda_{2} }}{\xi } + \xi \left( {1 + A cos\left( {\Omega t} \right)} \right)} \right\} - \left( {\lambda_{5} + \xi^{2} } \right)\emptyset_{s} \left( {\xi ,t} \right).$$

Solving fractional differential Eq. (19) by means of Laplace transform, we arrived at

20 $$\overline{\emptyset }_{s} \left( {\xi ,q} \right) = \frac{{\sqrt {\frac{2}{\pi }} \left\{ {\xi \left( {\frac{1}{q} + \frac{Aq}{{q^{2} + \omega^{2} }}} \right) + \frac{{\lambda_{2} }}{\xi }} \right\}\left( {q^{\alpha } + z_{0} } \right)^{2} }}{{\left( {z_{5} q^{2\alpha } + z_{6} q^{\alpha } + z_{7} } \right)}}.$$

Equation (20) is calculated on the basis of letting parameters as $\beta = \frac{1}{1 - \alpha },z_{0} = \alpha \beta ,z_{5} = \lambda_{3} \beta^{2} + \lambda_{4} \beta + \xi^{2} + \lambda_{5} ,$ $z_{6} = \lambda_{4} \alpha \beta^{2} + 2\xi^{2} z_{0} + 2\lambda_{5} z_{0} ,z_{7} = \xi^{2} z_{0}^{2} + \lambda_{5} z_{0}^{2}$. Applying inverse Fourier sine transform on Eq. (20) as:

21 $$\begin{aligned} \overline{\emptyset }\left( {y,q} \right) & = \left( {\frac{1}{q} + \frac{Aq}{{q^{2} + \omega^{2} }}} \right) - \frac{2}{\pi }\mathop \int \limits_{0}^{\infty } \frac{{\sin \left( {y\xi } \right)}}{\xi }\frac{1}{q}\left( {\frac{{q^{2\alpha } z_{8} + q^{\alpha } z_{9} + z_{10} }}{{q^{2\alpha } z_{5} + q^{\alpha } z_{6} + z_{7} }}} \right)d\xi - \frac{2}{\pi }\mathop \int \limits_{0}^{\infty } \frac{{sin\left( {y\xi } \right)}}{\xi }\frac{Aq}{{q^{2} + \omega^{2} }} \\ & \quad \times \;\left( {\frac{{q^{2\alpha } z_{8} + q^{\alpha } z_{9} + z_{10} }}{{q^{2\alpha } z_{5} + q^{\alpha } z_{6} + z_{7} }}} \right)d\xi + \frac{2}{\pi }\mathop \int \limits_{0}^{\infty } \frac{{sin\left( {y\xi } \right)}}{\xi }\left( {\frac{{\lambda_{2} \left( {q^{\alpha } + z_{0} } \right)^{2} }}{{q^{2\alpha } z_{5} + q^{\alpha } z_{6} + z_{7} }}} \right)d\xi . \\ \end{aligned}$$

The letting parameters for Eq. (21) are $z_{8} = z_{5} + \xi^{2} ,z_{9} = z_{6} + 2\xi^{2} z_{0} ,z_{10} = \xi^{2} z_{0}^{2}$. Writing Eq. (21) in to equivalent form by employing the procedures of infinite series as:

22 $$\begin{aligned} \overline{\emptyset }\left( {y,q} \right) & = \left( {\frac{1}{q} + \frac{Aq}{{q^{2} + \omega^{2} }}} \right) - \frac{2}{\pi }\mathop \int \limits_{0}^{\infty } \frac{{sin\left( {y\xi } \right)}}{\xi }\left( {\frac{1}{q} + \frac{Aq}{{q^{2} + \omega^{2} }}} \right)\left\{ {\frac{{z_{8} }}{{z_{7} }}} \right.\mathop \sum \limits_{{{\mathbb{R}}_{0} = 0}}^{\infty } \frac{{\left( { - \frac{{z_{5} }}{{z_{7} }}} \right)^{{{\mathbb{R}}_{0} }} }}{{{\mathbb{R}}_{0} !}}\mathop \sum \limits_{{{\mathbb{R}}_{1} = 0}}^{\infty } \frac{{\left( { - \frac{{z_{6} }}{{z_{5} }}} \right)^{{{\mathbb{R}}_{1} }} }}{{{\mathbb{R}}_{1} !}} \\ & \quad \times \;\frac{{\Gamma \left( {{\mathbb{R}}_{0} + 1} \right)\Gamma \left( {{\mathbb{R}}_{0} + 1} \right)}}{{q^{{\alpha {\mathbb{R}}_{1} - 2\alpha {\mathbb{R}}_{0} - 2\alpha }} \Gamma \left( {{\mathbb{R}}_{0} - {\mathbb{R}}_{1} + 1} \right)}} + \frac{{z_{9} }}{{z_{7} }}\mathop \sum \limits_{{{\mathbb{R}}_{2} = 0}}^{\infty } \frac{{\left( { - \frac{{z_{5} }}{{z_{7} }}} \right)^{{{\mathbb{R}}_{2} }} }}{{{\mathbb{R}}_{2} !}}\mathop \sum \limits_{{{\mathbb{R}}_{3} = 0}}^{\infty } \frac{{\left( { - \frac{{z_{6} }}{{z_{5} }}} \right)^{{{\mathbb{R}}_{3} }} }}{{{\mathbb{R}}_{3} !}}\frac{{\Gamma \left( {{\mathbb{R}}_{2} + 1} \right)\Gamma \left( {{\mathbb{R}}_{2} + 1} \right)}}{{q^{{\alpha {\mathbb{R}}_{3} - 2\alpha {\mathbb{R}}_{2} - \alpha }} \Gamma \left( {{\mathbb{R}}_{2} - {\mathbb{R}}_{3} + 1} \right)}} \\ & \quad + \;\frac{{z_{10} }}{{z_{7} }}\mathop \sum \limits_{{{\mathbb{R}}_{4} = 0}}^{\infty } \frac{{\left( { - \frac{{z_{5} }}{{z_{7} }}} \right)^{{{\mathbb{R}}_{4} }} }}{{{\mathbb{R}}_{4} !}}\mathop \sum \limits_{{{\mathbb{R}}_{5} = 0}}^{\infty } \frac{{\left( { - \frac{{z_{6} }}{{z_{5} }}} \right)^{{{\mathbb{R}}_{5} }} }}{{{\mathbb{R}}_{5} !}}\left. {\frac{{\Gamma \left( {{\mathbb{R}}_{4} + 1} \right)\Gamma \left( {{\mathbb{R}}_{4} + 1} \right)}}{{q^{{\alpha {\mathbb{R}}_{5} - 2\alpha {\mathbb{R}}_{4} }} \Gamma \left( {{\mathbb{R}}_{4} - {\mathbb{R}}_{5} + 1} \right)}}} \right\}d\xi + \frac{{2\lambda_{2} }}{\pi }\mathop \int \limits_{0}^{\infty } \frac{{\sin \left( {y\xi } \right)}}{\xi }\left\{ {\frac{1}{{z_{7} }}\mathop \sum \limits_{{{\mathbb{R}}_{0} = 0}}^{\infty } \frac{1}{{{\mathbb{R}}_{0} !}}} \right. \\ & \quad \times \;\left( { - \frac{{z_{5} }}{{z_{7} }}} \right)^{{{\mathbb{R}}_{0} }} \mathop \sum \limits_{{{\mathbb{R}}_{1} = 0}}^{\infty } \frac{{\left( { - \frac{{z_{6} }}{{z_{5} }}} \right)^{{{\mathbb{R}}_{1} }} }}{{{\mathbb{R}}_{1} !}}\frac{{\Gamma \left( {{\mathbb{R}}_{0} + 1} \right)\Gamma \left( {{\mathbb{R}}_{0} + 1} \right)}}{{q^{{\alpha {\mathbb{R}}_{1} - 2\alpha {\mathbb{R}}_{0} - 2\alpha }} \Gamma \left( {{\mathbb{R}}_{0} - {\mathbb{R}}_{1} + 1} \right)}} + \frac{{2z_{0} }}{{z_{7} }}\mathop \sum \limits_{{{\mathbb{R}}_{2} = 0}}^{\infty } \frac{{\left( { - \frac{{z_{5} }}{{z_{7} }}} \right)^{{{\mathbb{R}}_{2} }} }}{{{\mathbb{R}}_{2} !}}\mathop \sum \limits_{{{\mathbb{R}}_{3} = 0}}^{\infty } \frac{{\left( { - \frac{{z_{6} }}{{z_{5} }}} \right)^{{{\mathbb{R}}_{3} }} }}{{{\mathbb{R}}_{3} !}} \\ & \quad \times \;\frac{{\Gamma \left( {{\mathbb{R}}_{2} + 1} \right)\Gamma \left( {{\mathbb{R}}_{2} + 1} \right)}}{{q^{{\alpha {\mathbb{R}}_{3} - 2\alpha {\mathbb{R}}_{2} - \alpha }} \Gamma \left( {{\mathbb{R}}_{2} - {\mathbb{R}}_{3} + 1} \right)}} + \frac{{z_{0}^{2} }}{{z_{7} }}\mathop \sum \limits_{{{\mathbb{R}}_{4} = 0}}^{\infty } \frac{{\left( { - \frac{{z_{5} }}{{z_{7} }}} \right)^{{{\mathbb{R}}_{4} }} }}{{{\mathbb{R}}_{4} !}}\mathop \sum \limits_{{{\mathbb{R}}_{5} = 0}}^{\infty } \frac{{\left( { - \frac{{z_{6} }}{{z_{5} }}} \right)^{{{\mathbb{R}}_{5} }} }}{{{\mathbb{R}}_{5} !}}\left. {\frac{{\Gamma \left( {{\mathbb{R}}_{4} + 1} \right)\Gamma \left( {{\mathbb{R}}_{4} + 1} \right)}}{{q^{{\alpha {\mathbb{R}}_{5} - 2\alpha {\mathbb{R}}_{4} }} \Gamma \left( {{\mathbb{R}}_{4} - {\mathbb{R}}_{5} + 1} \right)}}} \right\}d\xi . \\ \end{aligned}$$

Inverting Eq. (22) by means of Laplace transform, we get

23 $$\begin{aligned} \emptyset \left( {y,t} \right) & = \left( {1 + Acos\left( {\omega t} \right)} \right) - \frac{2}{\pi }\mathop \int \limits_{0}^{\infty } \frac{{sin\left( {y\xi } \right)}}{\xi }\mathop \int \limits_{0}^{t} \left( {1 + Acos\omega \left( {t - z} \right)} \right)\left\{ {\frac{{z_{8} }}{{z_{7} }}\mathop \sum \limits_{{{\mathbb{R}}_{0} = 0}}^{\infty } \frac{{\left( { - \frac{{z_{5} }}{{z_{7} t^{2\alpha } }}} \right)^{{{\mathbb{R}}_{0} }} }}{{{\mathbb{R}}_{0} !}}\mathop \sum \limits_{{{\mathbb{R}}_{1} = 0}}^{\infty } \frac{{\left( { - \frac{{z_{6} t^{\alpha } }}{{z_{5} }}} \right)^{{{\mathbb{R}}_{1} }} }}{{{\mathbb{R}}_{1} !}}} \right. \\ & \quad \times \;\frac{{\Gamma \left( {{\mathbb{R}}_{0} + 1} \right)\Gamma \left( {{\mathbb{R}}_{0} + 1} \right)t^{ - 2\alpha - 1} }}{{\Gamma \left( {\alpha {\mathbb{R}}_{1} - 2\alpha {\mathbb{R}}_{0} - 2\alpha } \right)\Gamma \left( {{\mathbb{R}}_{0} - {\mathbb{R}}_{1} + 1} \right)}} + \frac{{z_{9} }}{{z_{7} }}\mathop \sum \limits_{{{\mathbb{R}}_{2} = 0}}^{\infty } \frac{{\left( { - \frac{{z_{5} }}{{z_{7} t^{2\alpha } }}} \right)^{{{\mathbb{R}}_{2} }} }}{{{\mathbb{R}}_{2} !}}\frac{{\Gamma \left( {{\mathbb{R}}_{2} + 1} \right)\Gamma \left( {{\mathbb{R}}_{2} + 1} \right)t^{ - \alpha - 1} }}{{\Gamma \left( {\alpha {\mathbb{R}}_{3} - 2\alpha {\mathbb{R}}_{2} - \alpha } \right)\Gamma \left( {{\mathbb{R}}_{2} - {\mathbb{R}}_{3} + 1} \right)}} \\ & \quad \times \;\mathop \sum \limits_{{{\mathbb{R}}_{3} = 0}}^{\infty } \frac{{\left( { - \frac{{z_{6} t^{\alpha } }}{{z_{5} }}} \right)^{{{\mathbb{R}}_{3} }} }}{{{\mathbb{R}}_{3} !}} + \frac{{z_{10} }}{{z_{7} }}\mathop \sum \limits_{{{\mathbb{R}}_{4} = 0}}^{\infty } \frac{{\left( { - \frac{{z_{5} }}{{z_{7} t^{2\alpha } }}} \right)^{{{\mathbb{R}}_{4} }} }}{{{\mathbb{R}}_{4} !}}\mathop \sum \limits_{{{\mathbb{R}}_{5} = 0}}^{\infty } \frac{{\left( { - \frac{{z_{6} t^{\alpha } }}{{z_{5} }}} \right)^{{{\mathbb{R}}_{5} }} }}{{{\mathbb{R}}_{5} !}}\left. {\frac{{\Gamma \left( {{\mathbb{R}}_{4} + 1} \right)\Gamma \left( {{\mathbb{R}}_{4} + 1} \right)t^{ - 1} }}{{\Gamma \left( {\alpha {\mathbb{R}}_{5} - 2\alpha {\mathbb{R}}_{4} } \right)\Gamma \left( {{\mathbb{R}}_{4} - {\mathbb{R}}_{5} + 1} \right)}}} \right\}d\xi dz \\ & \quad + \;\frac{{2\lambda_{2} }}{\pi }\mathop \int \limits_{0}^{\infty } \frac{{\sin \left( {y\xi } \right)}}{\xi }\left\{ {\frac{1}{{z_{7} }}\mathop \sum \limits_{{{\mathbb{R}}_{0} = 0}}^{\infty } \frac{{\left( { - \frac{{z_{5} }}{{z_{7} t^{2\alpha } }}} \right)}}{{{\mathbb{R}}_{0} !}}^{{{\mathbb{R}}_{0} }} \mathop \sum \limits_{{{\mathbb{R}}_{1} = 0}}^{\infty } \frac{{\left( { - \frac{{z_{6} t^{\alpha } }}{{z_{5} }}} \right)^{{{\mathbb{R}}_{1} }} }}{{{\mathbb{R}}_{1} !}}\frac{{\Gamma \left( {{\mathbb{R}}_{0} + 1} \right)\Gamma \left( {{\mathbb{R}}_{0} + 1} \right)t^{ - 2\alpha - 1} }}{{\Gamma \left( {\alpha {\mathbb{R}}_{1} - 2\alpha {\mathbb{R}}_{0} - 2\alpha } \right)\Gamma \left( {{\mathbb{R}}_{0} - {\mathbb{R}}_{1} + 1} \right)}}} \right. \\ & \quad + \;\frac{{2z_{0} }}{{z_{7} }}\mathop \sum \limits_{{{\mathbb{R}}_{2} = 0}}^{\infty } \frac{{\left( { - \frac{{z_{5} }}{{z_{7} t^{2\alpha } }}} \right)^{{{\mathbb{R}}_{2} }} }}{{{\mathbb{R}}_{2} !}}\mathop \sum \limits_{{{\mathbb{R}}_{3} = 0}}^{\infty } \frac{{\left( { - \frac{{z_{6} t^{\alpha } }}{{z_{5} }}} \right)^{{{\mathbb{R}}_{3} }} }}{{{\mathbb{R}}_{3} !}}\frac{{\Gamma \left( {{\mathbb{R}}_{2} + 1} \right)\Gamma \left( {{\mathbb{R}}_{2} + 1} \right)t^{ - \alpha - 1} }}{{\Gamma \left( {\alpha {\mathbb{R}}_{3} - 2\alpha {\mathbb{R}}_{2} - \alpha } \right)\Gamma \left( {{\mathbb{R}}_{2} - {\mathbb{R}}_{3} + 1} \right)}} + \frac{{z_{0}^{2} }}{{z_{7} }}\mathop \sum \limits_{{{\mathbb{R}}_{4} = 0}}^{\infty } \frac{{\left( { - \frac{{z_{5} }}{{z_{7} t^{2\alpha } }}} \right)^{{{\mathbb{R}}_{4} }} }}{{{\mathbb{R}}_{4} !}} \\ & \quad \times \;\mathop \sum \limits_{{{\mathbb{R}}_{5} = 0}}^{\infty } \frac{{\left( { - \frac{{z_{6} t^{\alpha } }}{{z_{5} }}} \right)^{{{\mathbb{R}}_{5} }} }}{{{\mathbb{R}}_{5} !}}\left. {\frac{{\Gamma \left( {{\mathbb{R}}_{4} + 1} \right)\Gamma \left( {{\mathbb{R}}_{4} + 1} \right)t^{ - 1} }}{{\Gamma \left( {\alpha {\mathbb{R}}_{5} - 2\alpha {\mathbb{R}}_{4} } \right)\Gamma \left( {{\mathbb{R}}_{4} - {\mathbb{R}}_{5} + 1} \right)}}} \right\}d\xi . \\ \end{aligned}$$

Equation (23) is an analytical solution of hyperbolic heat transfer equations in longitudinal fin that satisfies the imposed conditions.

## Results and concluding discussion

This portion is dedicated for physical insights and practical aspects from heat transfer of longitudinal fin. The fractional modeling of Fourier and non-Fourier heat transfer of longitudinal fin via non-singular fractional approach is depicted through various types of graphs. The graphical illustration is based on supplying constraint to have significant heat transfer from longitudinal fins. The configuration of longitudinal fin in terms of one dimension is developed for the mathematical model of parabolic and hyperbolic heat transfer equations in which several physical parameters are discussed within the suitable values. By considering the Fourier and non-Fourier heat transfer from longitudinal fin, the mathematical techniques of Fourier Sine and Laplace transforms have proved the better conduction to diffuse the heat away from cooled aspects. An analytic approach is tackled for handling the governing equation through special functions for the fractionalized parabolic and hyperbolic heat transfer equations in longitudinal fin. For the sake of comparative analysis of parabolic verses hyperbolic heat conduction of fin temperature, we depicted the distinct graphical illustrations; for instance, 2-dimensional graph, bar chart graph, contour graphs, heat graph, 3-dimensional graphs and column graphs on for the variants of different rheological impacts of longitudinal fin. In short, the following outcomes have been achieved on the basis of different variants in rheological parameters as:

### Dynamical aspects of relaxation time for parabolic verses hyperbolic heat conduction

**Figure 3:**
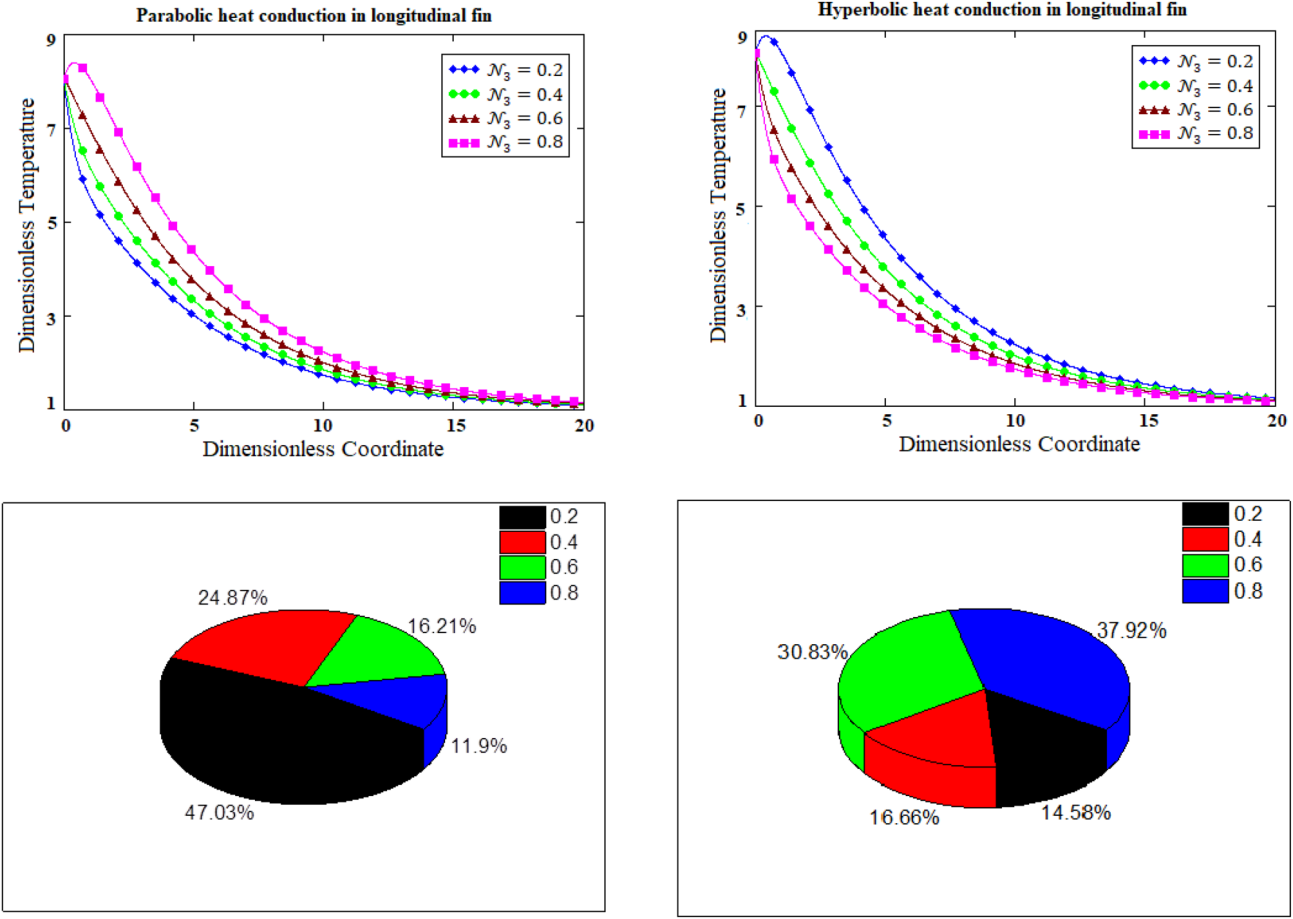
Comparative graphs of parabolic verses hyperbolic heat conduction for the variants of relaxation time based on 2-dimensional and bar chart graphs.

Relaxation time is an important parameter that can determine parabolic and hyperbolic performance of heat conduction significantly. This is because when thermoelectric properties of certain materials are needed then relaxation time is generally employed. Figure 3 is prepared for the comparative graphical illustration of parabolic verses hyperbolic heat conduction for the variants of relaxation time based on 2-dimensional and bar chart graphs. It is observed that temperature distribution of parabolic heat conduction of fin in 2-dimensional graph has controversial trend in comparison with temperature distribution of hyperbolic heat conduction of fin. Whilst, bar chart graphs as shown in Fig. 3 shows the reversal behavior of temperature distribution. In brevity, temperature distribution of parabolic heat conduction of fin in bar chart graph is dominant than temperature distribution of hyperbolic heat conduction of fin.

### Dynamical aspects of frequency for parabolic verses hyperbolic heat conduction

**Figure 4:**
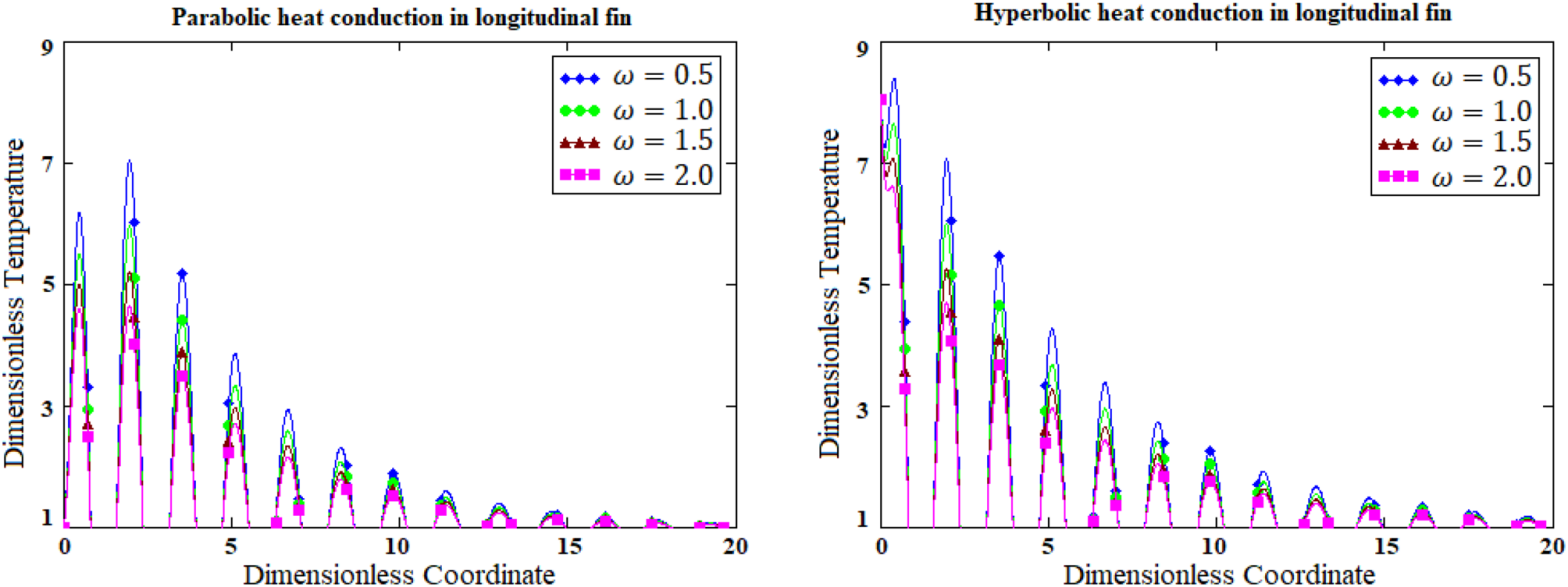
Comparative graphs of parabolic verses hyperbolic heat conduction for the variants of frequency of the base temperature based on 2-dimensional graph.

The higher frequency always relates a higher energy either in parabolic heat conduction or in hyperbolic heat conduction. Figure 4 is prepared for knowing the dynamical role of frequency of parabolic heat conduction and hyperbolic heat conduction separately. It is observed that increasing values of frequency have generated peak oscillations in hyperbolic heat conduction in comparison with parabolic heat conduction.

### Dynamical aspects of amplitude for parabolic verses hyperbolic heat conduction

**Figure 5:**
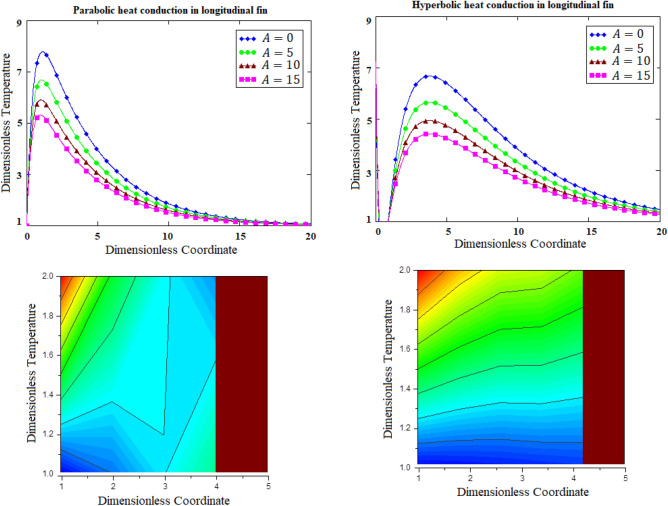
Comparative graphs of parabolic verses hyperbolic heat conduction for the variants of amplitude of the base temperature based on 2-dimensional and contour graphs.

**Figure 6:**
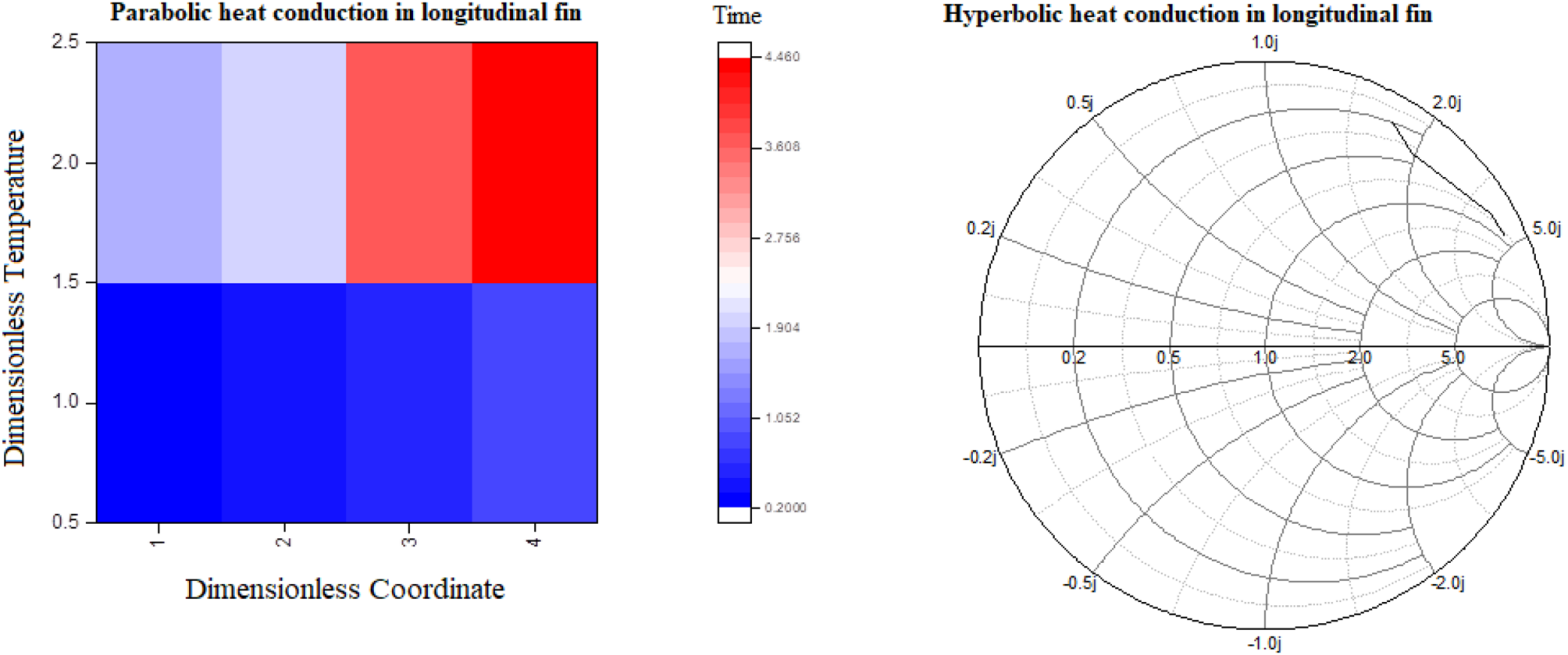
Heat graph of parabolic heat conduction for the variants of time and Smith graph for hyperbolic heat conduction for scatterings of temperature distribution.

**Figure 7:**
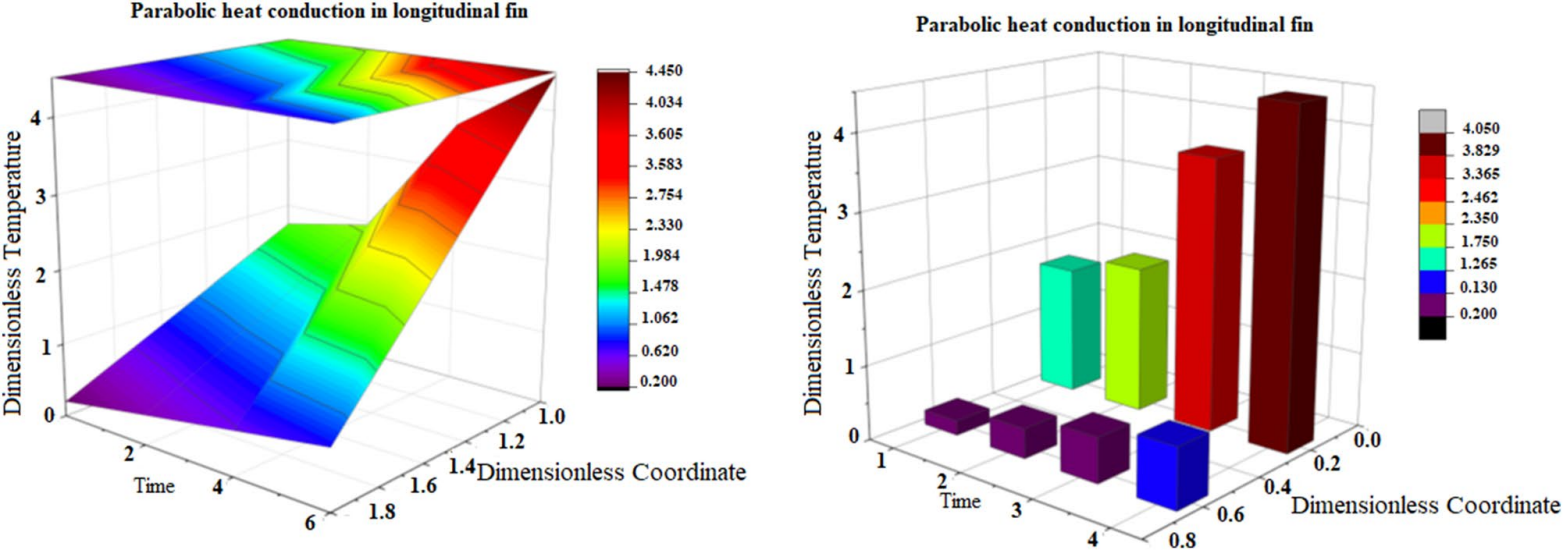
3-dimensional and column graphs of parabolic heat conduction for the variants of fractional parameter for scatterings of temperature distribution with respect to time in seconds.

The comparative graphs in terms of two-dimensional and contour graphs of parabolic verses hyperbolic heat conduction for the variants of amplitude of the base temperature have been prepared in Fig. 5. It is perceived that by increasing amplitude, the non-resistive trends have been disclosed by both parabolic as well as hyperbolic heat conduction. This is many be due to the fact that when parabolic or hyperbolic heat conduction is subjected for incremental amplitude then amplitude response becomes aperiodic. Additionally, Fig. 6 is depicted for heat graph of parabolic heat conduction for the variants of time and Smith graph for hyperbolic heat conduction for scatterings of temperature distribution. Here, heat graph of parabolic heat conduction has shown phase transitions on the basis of increasing time. While, Smith graph for hyperbolic heat conduction is also observed for temperature distribution. In exaggeration, 3-dimensional and column graphs of parabolic heat conduction for the variants of fractional parameter for scatterings of temperature distribution have been prepared in Fig. 7. It is observed that fractional parameter has significance rise in temperature distribution.

### Fractional and classical comparative analysis of parabolic verses hyperbolic heat conduction

**Figure 8:**
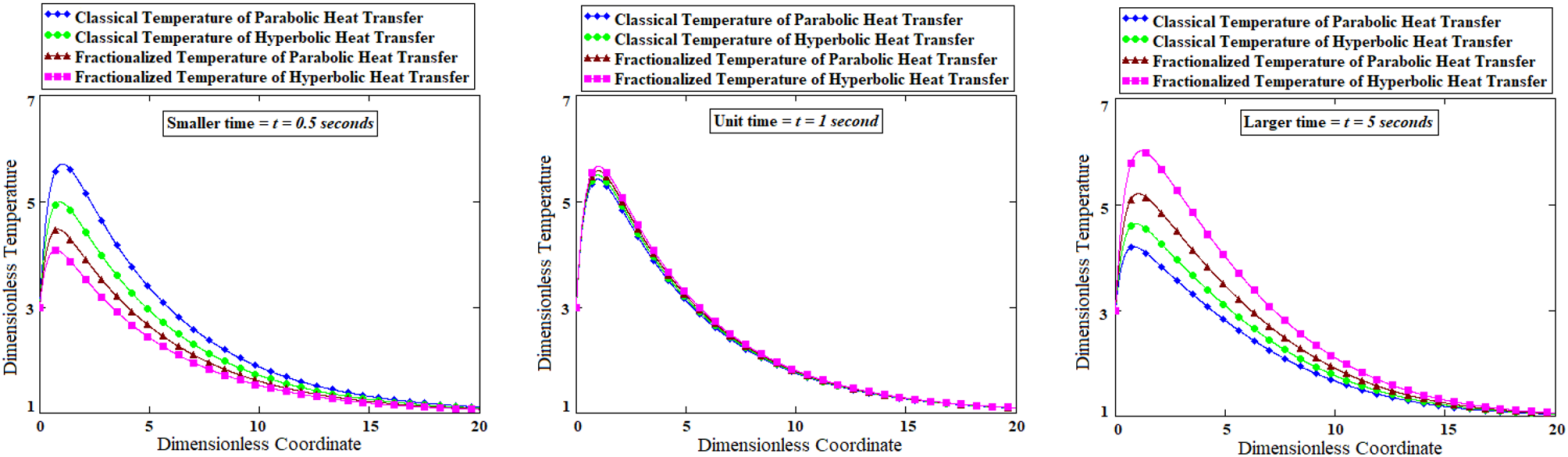
Comparative graphs of parabolic verses hyperbolic heat conduction for the classical and fractional approaches based on three different times.

The comparison of classical and fractional methods measures the closeness of agreement for integer and non-integer differentiations. Such comparison leads to estimate inaccuracy and accuracy of investigated solutions among imposed classical and fractional methods. Figure 8 is prepared for the classical and fractionalized temperature with parabolic and hyperbolic heat transfer at three diffreent times (smaller, unit and larger times). The four types of analytical solutions have been compared namely (i) classical temperature of parabolic heat transfer, (ii) classical temperature of hyperbolic heat transfer, (iii) fractional temperature of parabolic heat transfer, and (iv) fractionalzed temperature of hyperbolic heat transfer. For smaller time *t* = 0.5 s, fractionalzed temperature of hyperbolic heat transfer is switer than (i) classical temperature of parabolic heat transfer, (ii) classical temperature of hyperbolic heat transfer, (iii) fractional temperature of parabolic heat transfer. On the contrary, for larger time *t* = 5 s, fractionalzed temperature of hyperbolic heat transfer moves faster than (i) classical temperature of parabolic heat transfer, (ii) classical temperature of hyperbolic heat transfer, (iii) fractional temperature of parabolic heat transfer. For the sake of phyical sigficance, all the models have coincidence temperature at unit time *t* = 1 s.

## Conclusion

In this study, the fractionalized analytical solutions of parabolic and hyperbolic heat transfer based on temperature distribution have been obtained by employing integral transforms as shown in Fig. 2. The results for temperature profiles have decalred physical insights and practical aspects from heat transfer of longitudinal fin. The different variants in rheological parameters have been shown various investigations, such investigations are enumerated as:

(i) The temperature distribution of parabolic heat conduction of fin has controversial trend in comparison with temperature distribution of hyperbolic heat conduction of fin.
(ii) Increasing values of frequency have generated peak oscillations in hyperbolic heat conduction in comparison with parabolic heat conduction.
(iii) Either parabolic or hyperbolic heat conduction is subjected for incremental amplitude then amplitude response becomes aperiodic.
(iv) The comparison of classical and fractional methods measures the closeness to estimate inaccuracy and accuracy of investigated solutions.
